# Morphologic Characters of the Rostrum in Two Weevils, *Eucryptorrhynchus scrobiculatus* Motschulsky and *E. brandti* Harold (Coleoptera: Curculionidae: Cryptorrhychinae)

**DOI:** 10.3390/insects14010071

**Published:** 2023-01-11

**Authors:** Ganyu Zhang, Ruihong Sun, Huijuan Li, Junbao Wen

**Affiliations:** 1 Shandong Institute of Pomology, Shandong Academy of Agricultural Sciences, Tai’an 271000, China; 2 Key Laboratory of National Forestry and Grassland Administration, Beijing Forestry University, Beijing 100083, China

**Keywords:** rostrum, *Eucryptorrhynchus scrobiculatus*, *Eucryptorrhynchus brandti*, structure, oviposition, coexistence

## Abstract

**Simple Summary:**

*Eucryptorrhynchus scrobiculatus* and *E. brandti* are boring weevils of *Ailanthus altissima* (tree-of-heaven) and coexist on the same host *A. altissima*. In previous studies, through behavioral observation, we learned that, during the oviposition process, these weevils need to use their rostra to excavate an oviposition hole. However, the specific morphology of the rostra of the two weevils and the egg-laying mechanism during the oviposition process currently remain unknown. The morphological analysis attempts to link biological structure and function to specific environmental or behavioral characteristics, and this method has become an indispensable tool in the process of elucidating and interpreting patterns. Therefore, the morphologic characteristics of the rostra in *E. scrobiculatus and E. brandti* were examined and compared by scanning electron microscopy and micro-CT. This study not only plays an important role in exploring the excavating mechanism during oviposition of *E. scrobiculatus and E. brandti*, but also provides new insight for explaining the coexistence of two weevil species in the same host.

**Abstract:**

(1) *Eucryptorrhynchus scrobiculatus* and *E. brandti* (Coleoptera: Curculionidae: Cryptorrhychinae) are both pests of *Ailanthus altissima*, found in China. During ovipositing, gravid females of the two weevils need to excavate a cavity in the oviposition substrate with their rostrum, while their oviposition sites are different. (2) In this study, to explore the boring mechanism of *E. scrobiculatus and E. brandti* during ovipositing, the morphologic characters of the rostra of two weevils were studied in detail by scanning electron microscopy and micro-CT. (3) Their rostra appear similar, but the rostrum surface of *E. scrobiculatus* is rougher than that of *E. brandti*; their fine structures of rostrum and sensilla distribution are similar, but the sensilla twig basiconica 3 is distributed at the apex of labial palpus in *E. brandti* females, while not at the apex of labial palpus in *E. scrobiculatus* females; their rostra are hollow and their cuticle thickness is constantly changing, but the proportion of the whole rostrum tube cuticle in *E. scrobiculatus* is significantly larger than that of *E. brandti.* The above structural differences make *E. scrobiculatus* more conducive to oviposition in the soil and *E. brandti* more conducive to oviposition in the trunk of *A. altissima*. (4) Overall, this study not only plays an important role in exploring the excavating mechanism during the oviposition of the two weevils, but also provides new insights into the coexistence of two weevil species on the same host *A. altissima*.

## 1. Introduction

Weevils are a type of beetle belonging to the superfamily Curculionoidea. They are one of the most diverse groups in the existing biological family, with about 60,000 described species [1,2].

In insects, the structure and function of mouthparts and their relationship with feeding strategies have attracted increasing research attention. The morphology of insect mouthparts, as well as the adaptation characteristics of host localization, feeding, and oviposition related to mouthparts morphology, have been extensively studied. For example, the principal morphological adaptations of species of *Oxyporus* (Coleoptera: Staphylinidae) to fungal feeding involve modifications of the mandibles of adults and larvae; labial palpi and labrum of adults; and maxillae of larvae [3]. Studies show that a detailed observation of the cuticular structure of the mouthpart of *Ips acuminatus* (Coleoptera: Curculionidae: Scolytinae) using field emission scanning electron microscopy (FESEM) demonstrated its possible implication to act as an external carrier of pathogenic microorganism [4]. *Doubledaya bucculenta* Lewis (Coleoptera, Erotylidae, Trypanosomidae) shows an asymmetry in the mandibles of females compared to males, which helps females to dig holes between hard bamboo nodes when laying eggs [5]. In weevils, the mouthparts are situated at the end of a rostrum and vary in length. The mouthparts of weevils are mainly involved in the mechanics of feeding, processing, and manipulating food. However, in some species, it has been extended to other functions, in addition to the traditional feeding function, the rostrum of the weevils can also be used to prepare oviposition sites [6,7,8]. Most weevils use a long rostrum as a sclerotized ovipositor, which can dig a hole and place an egg in secret or other inaccessible places for insects (due to the structural characteristics of plants) [1]. Using rostra to prepare oviposition sites can help avoid the physical defense of plants (shells and spines) and larvae drying, as well as initiating and maintaining the key adaptation to host attachment [1]. The appearance of the rostrum allows the weevil group to feed and lay eggs in almost all plant tissues [2,9,10,11].

*Eucryptorrhynchus scrobiculatus* Motschulsky and *E. brandti* Harold (Coleoptera: Curculionidae: Cryptorrhychinae) are widely distributed in China [12,13,14,15]. These two weevilshave a high specificity to *Ailanthus altissima* (Mill.) Swingle (tree-of-heaven) (Simaroubaceae) and its variety *A. altissima* var, cause serious damage to forestry in China at the larval stage [13,14,15,16,17,18,19]. *E. scrobiculatus* larvae damage the root of *A. altissima*, *E. brandti* larvae damage the trunk of *A. altissima* [14,15,16]. Previous studies found that the difference in the feeding position of the two weevil larvae was related to the oviposition sites of the two weevils [20]. The oviposition sites of *E. scrobiculatus* and *E. brandti* are different. *E. scrobiculatus* females lay eggs in the soil near *A. altissima*, while *E. brandti* females lay eggs in the trunk of *A. altissima* [20,21]. The two weevils utilize different oviposition sites and these differences in habitat use may reduce the competition for resources between species during the larval period, thus facilitating their coexistence in *A. altissima* [20]. Studies on the oviposition behavior of two weevils show that both weevils need to excavate an oviposition cavity with their rostrum before laying eggs [20,21]. However, the specific morphology and structure of the rostrum of the two weevils and the excavation mechanism during the oviposition process are currently unknown. Therefore, the focus of this study is to explore the fine structure of the rostra of *E. scrobiculatus* and *E. brandti*, provide a detailed morphology comparison of the rostra to understand the functional role of these structures in the oviposition process, and explore the reasons for differences in the oviposition sites of the two weevils.

## 2. Materials and Methods

### 2.1. Insect Collecting

Adults of *E. scrobiculatus* and *E. brandti* were collected from the *A. altissima* forest in Xiaoxingdun village (38°51′ N, 106°31′ E), Ningxia Hui Autonomous, China. They were reared in plastic boxes with fresh branches of *A. altissima*.

### 2.2. Scanning Electron Microscopy

Twenty specimens of *E. scrobiculatus* females and twenty specimens of *E. brandti* females were put into a plastic bottle filled with 70% ethanol. Each weevil was taken out of the bottle one by one, and its head (with rostrum) and body were separated with tweezers and dissecting scissors. The dissected head was put into ultrapure water for ultrasonic cleaning, and the water was changed every 5 min for a total of 15 min. The cleaned heads were dehydrated with 30%, 50%, 70%, 80%, 90%, 95%, and 100% alcohol gradients for 15 min, which was repeated once in 100% ethanol. The mandibles, maxillae, and labium of the two weevils were dissected under a microscope (DM2500, Leica), and at the same time, the samples were pasted on the sample stage according to the required shooting angle with conductive glue. The stage with the samples attached was placed in a plastic box filled with silica gel desiccant and dried at room temperature. All samples were coated with a film of gold-palladium in an E-1010 sputter ion instrument (Hitachi, Tokyo, Japan). Then, the observation and photographs were taken under an S-3400N (Hitachi) scanning electron microscope at an accelerating voltage of 0.5–30 KV.

### 2.3. X-ray Micrograph

The rostrum was separated from the body using the same method as above. Five rostra of *E. scrobiculatus* females and five rostra of *E. brandti* females were put into a plastic bottle filled with 70% ethanol. The dissected rostra were fixed in Bouin solution, washed with 70% ethanol, and then dehydrated by alcohol gradient. The alcohol concentrations were 50%, 70%, 75%, 80%, 85%, 90%, 95%, 100% (3 times), with an interval of 10–15 min. Finally, the sample was dehydrated with acetone 3 times, each for 30 min. The prepared sample was placed under a 3D X-ray microscope (nanoVoxel-3000, Sanying Precision Instrument Co., Ltd., Tianjin, China) for shooting and scanning. The scanning parameters were as follows: voltage 60.0 kV, current 80.0 μmA, time 0.60 s.

### 2.4. Three-Dimensional Reconstruction

The target structure was reconstructed using the imported data analyzed by Avizo 9.0.1. The 916 micro-CT slices of *E. scrobiculatus* and 604 micro-CT slices of *E. brandti* were used to reconstruct rostrum tubes, respectively. These structures were trimmed, smoothed, and rendered with multiple viewers.

### 2.5. Data Analyses

The references for the classification of the sensilla were as follows [7,22,23,24,25]. The mean length and width of the sensillum were measured using ImageJ. Data analyses were performed using SPSS 25.0. One-way ANOVA and Tukey’s HSD tests (*p* < 0.05) were used to compare the proportion of the rostrum tube cuticle. Graphs were drawn with GraphPad Prism 9, scales and arrows on the images were marked using Photoshop 2021.

## 3. Results

### 3.1. Overall Structure of E. scrobiculatus and E. brandti Rostrum

**Figure 1:**
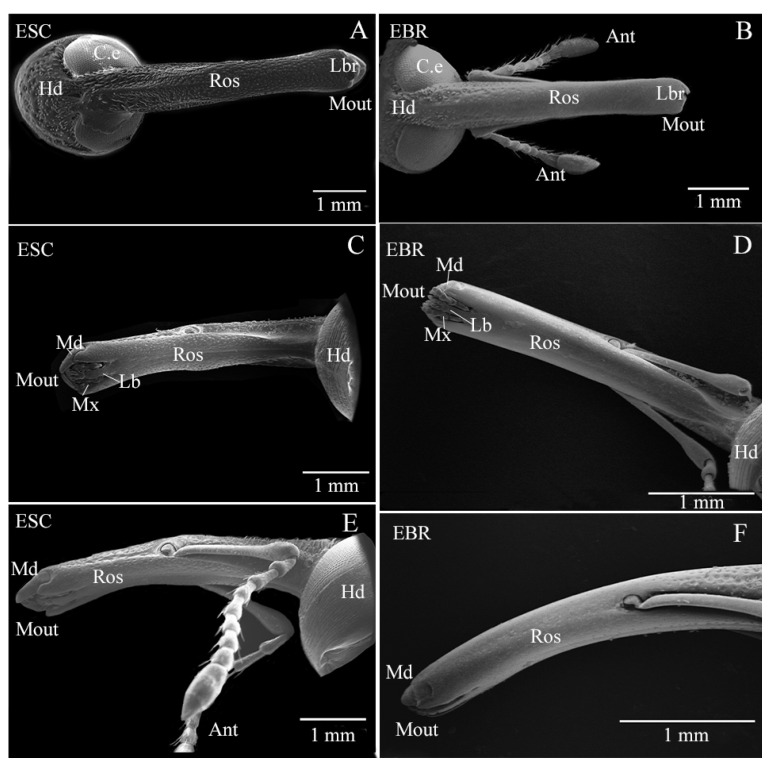
Scanning electron micrographs showing the overall appearance of *Eucryptorrhynchus scrobiculatus* (ESC) and *E. brandti* (EBR) rostrum. (**A**,**C**,**E**) the overall appearance of *E. scrobiculatus* rostrum (the dorsal view, the ventral view and the lateral view, respectively); (**B**,**D**,**F**) the overall appearance of *E. brandti* rostrum (the dorsal view, the ventral view and the lateral view, respectively). Hd, Head; C.e, Compound eyes; Ros, Rostrum; Ant, Antenna; Mout, Mouthpart; Lbr, Labrum; Md, Mandible; Mx, Maxillae; Lab, Labium.

Scanning electron microscope observation showed that although in terms of overall structure the rostra morphology of *E. scrobiculatus* and *E. brandti* looked similar, there were some differences. The downward curvature of the entire rostrum of *E. scrobiculatus* female is inconsistent. The downward curvature of the rostrum from the antennal fossa to the tip is larger than that from the antennal fossa to the head. The rostrum surface of *E. scrobiculatus* female is rough and has convex edges and grooves, covering a large number of setae and pores (Figure 1A,C,E). The entire rostrum of *E. brandti* female smoothly curves downward at the same curvature. The rostrum surface is relatively smooth, with no clearly defined convex edges or grooves, and relatively few setae and pores (Figure 1B,D,F). The mouthparts of *E. scrobiculatus and E. brandti* are located at the tip of the rostrum extended outwards from the head. The mouthparts of both weevils consist of a labrum (Lbr), a pair of mandibles (Md), a pair of maxillae (Mx) and a labium (Lb) (Figure 1). From Figure 1C,D, we can see the difference between the tips of the two weevil’s rostra. The maxillae (Mx) and labium (Lb) of *E. scrobiculatus* are encased by the mandibles and shorter than the mandibles, while the maxillae (Mx) and labium (Lb) of *E. brandti* are not completely enclosed by mandibles; some parts are longer than the mandibles and are exposed to the outside of them. Therefore, the fine structures of the maxillae and labium of the two weevils were compared in the following study.

### 3.2. The Apex Structure of E. scrobiculatus and E. brandti Rostrum

#### 3.2.1. Maxillae, Mx

**Figure 2:**
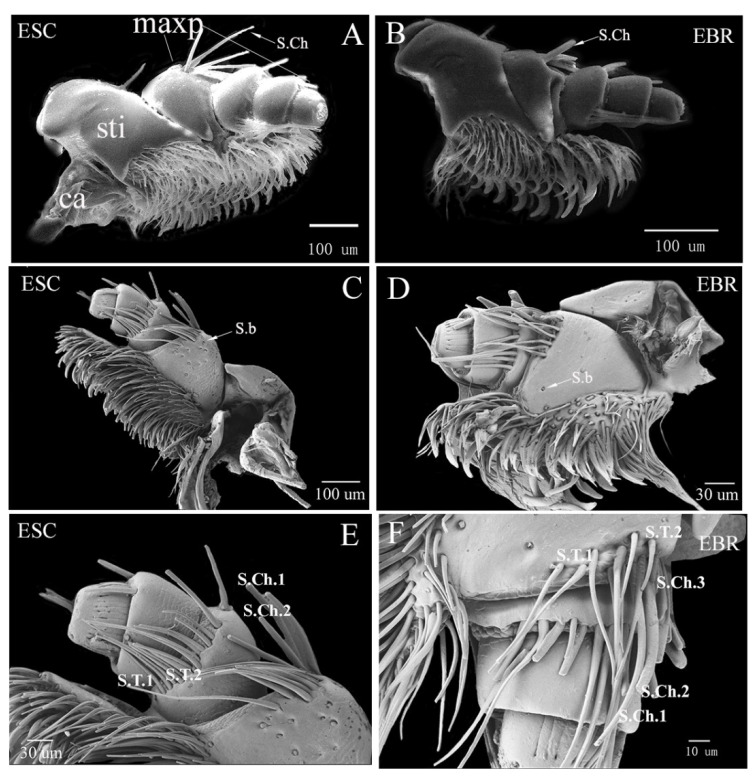
Scanning electron micrographs showing the maxillae of *Eucryptorrhynchus scrobiculatus* (ESC) females and *E. brandti* (EBR) females. (**A**) The dorsal of *E. scrobiculatus* maxillae; (**B**) the dorsal of *E. brandti* maxillae; (**C**) the ventral of *E. scrobiculatus* maxillae; (**D**) the ventral of *E. brandti* maxillae; (**E**) the ventral of *E. scrobiculatus* maxillary palpi; (**F**) the ventral of *E. brandti* maxillary palpi. maxp, maxillary palpi; sti, stipe; ca, cardo; S.Ch, sensilla chaetica; S.b, sensilla basiconica; S.T.1-2, sensilla trichodea; S.Ch.1-3, sensilla chaetica.

The maxillae of *E. scrobiculatus* and *E. brandti* are located in the longitudinal cleft on both sides of the labium. There are a pair of segmented movable appendages. The maxillae can be divided into five parts: cardo, stipe, galea, laciniae, and maxillary palpi. The galea and laciniae covered with a large number of setae are attached to one side of the stipe. There are some differences in the number and distribution of sensors in the stipe for the two weevils. The stipe is a large sclerite with a smoother surface connected to the end of the cardo (Figure 2A). A small number of sensilla basiconica are distributed on the ventral of the stipe, and a few sensilla chaetica and sensilla trichodea are concentrated at the top. Depending on different length and width, in *E. scrobiculatus*, the sensilla trichodea can be divided into two types: S.T.1 and S.T.2, and the sensilla chaetica can be divided into two types: S.Ch.1 and S.Ch.2 (Figure 2E). In *E. brandti*, the sensilla trichodea can be divided into two types: ST1 and S.T.2, and the sensilla chaetica can be divided into three types: S.Ch.1, S.Ch.2 and S.Ch.3 (Figure 2F). The number of sensilla basiconica of *E. scrobiculatus* on the ventral stipe was higher than that of *E. brandti* (Figure 2C,D).

**Figure 3:**
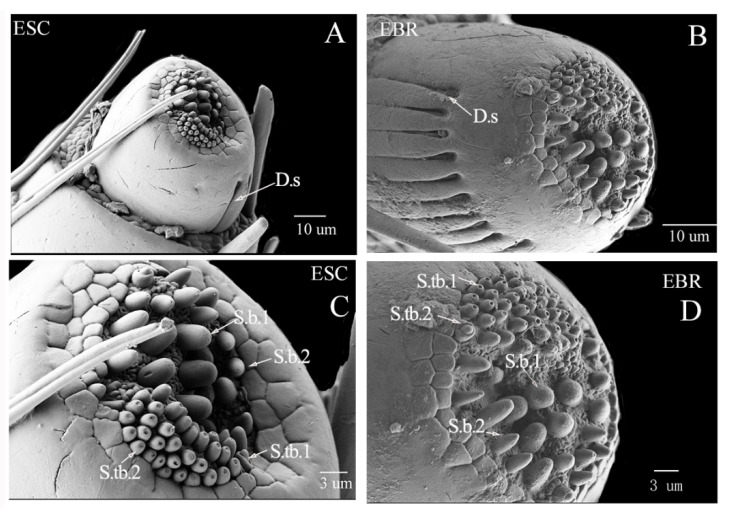
Scanning electron micrographs showing the first section of maxillary palpi of *Eucryptorrhynchus scrobiculatus* (ESC) females and *E. brandti* (EBR) females. (**A**,**C**) the first section of maxillary palpi of *E. scrobiculatus*; (**B**,**D**) the first section of maxillary palpi of *E. brandti.* Ds, digitiform sensilla; S.b.1-2, sensilla basiconica; S.tb.1-2, sensilla twig basiconica.

The maxillary palpi are segmental structures found in the middle of the outer edge of the stipe. Because the last section is embedded in the stipe, there are only three sections of the maxillary palpi from the ventral side, but there are four sections when viewed from the dorsal side (Figure 2A–D). The first section is round-shaped, with several digitiform sensilla (D.s) distributed on the upper ventral surface, but the backside is smooth (Figure 3A,B). The ends of the truncated mandibular whiskers are sunken and cracked, and contain many sensory papillae of different sizes. These sensors can be divided into two sensilla basiconica (S.b.1 and S.b.2) and three sensilla twig basiconica (S.tb.1–3) by types (Figure 3C,D). A small number of sensilla chaetica are distributed in the 2nd–4th segments of the maxillary palpi of the two weevils, but the lengths of the sensilla chaetica in the fourth segment of *E. scrobiculatus* and *E. brandti* are different. The length of the sensilla chaetica in *E. scrobiculatus* can be extended from the fourth section to the top of the third segment, and some are even close to the second segment, but the length of the sensilla chaetica in *E. brandti* can only be extended from the fourth section to half of the third segment (Figure 2A,B).

#### 3.2.2. Labium, Lb

**Figure 4:**
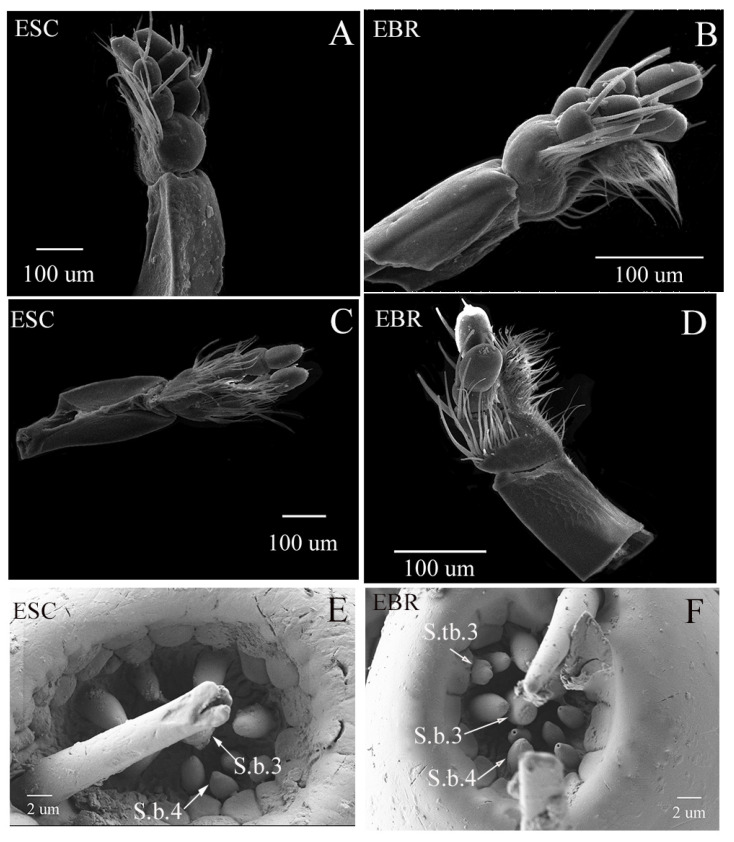
Scanning electron micrographs showing the labium of *Eucryptorrhynchus scrobiculatus* (ESC) females and *E. brandti* (EBR) females. (**A**) © ventral of *E. scrobiculatus* labium; (**B**) the ventral of *E. brandti* labium; (**C**) the dorsal of *E. scrobiculatus* labium; (**D**) the dorsal of *E. brandti* labium. (**E**) the apex of labial palpus of *E. scrobiculatus*; (**F**) the apex of labial palpus of *E. brandti.* S.b.3-4, sensilla basiconica; S.tb.3, sensilla twig basiconica.

The labium is located on the ventral side of the mouthparts. The abdominal side is smooth and the inner side has a distinct ridge. The labium has a pair of labial palpus, and each labial palpus has three segments (Figure 4). The overall structure of the labium of the two weevils appears similar, and the type and distribution of the sensilla are roughly the same, except that different types of sensilla are found at the end of the first section of the labial palpus. In *E. scrobiculatus*, there are two sensors (S.b.3 and S.b.4) distributed at this position, but in *E. brandti*, there are three sensors (S.b.3, S.b.4 and S.tb3) distributed, and the sensor S.tb.3 is found only at the apex of labial palpus of *E. brandti* females (Figure 4E,F).

### 3.3. Exoskeleton Morphology of the Rostrum

**Figure 5:**
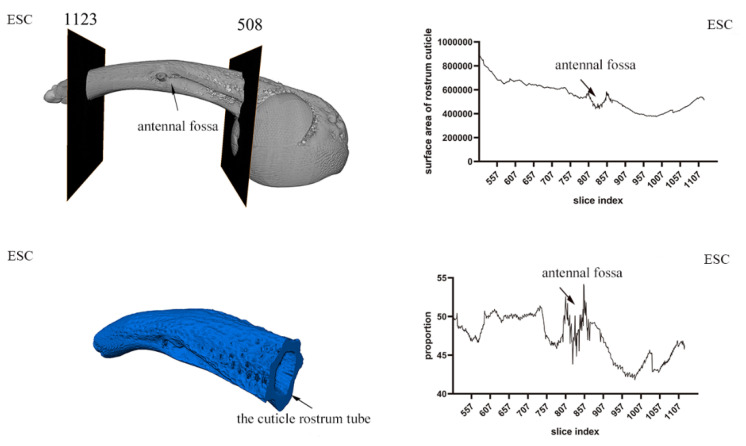
The rostral cuticle thickness of *Eucryptorrhynchus scrobiculatus* (ESC) females. The left is the three-dimensional reconstruction structure of the rostrum tube by micro-CT; the right is the change of the thickness of the rostral cuticle.

**Figure 6:**
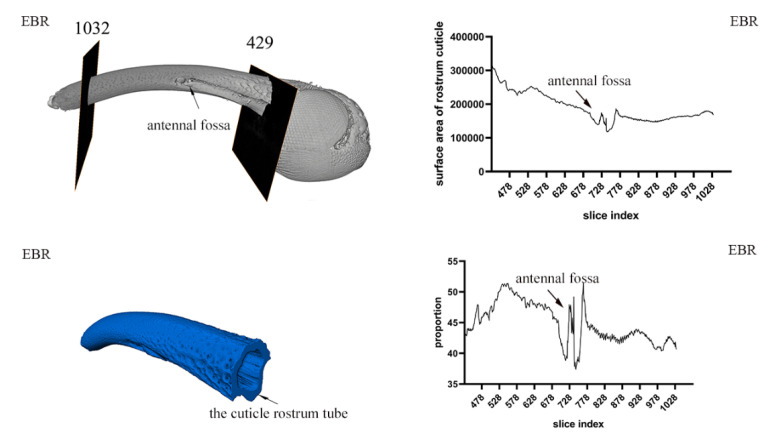
The rostral cuticle thickness of *Eucryptorrhynchus brandti* (EBR) females. The left is the three-dimensional reconstruction structure of the rostrum tube by micro-CT; the right is the change of the thickness of the rostral cuticle.

**Figure 7:**
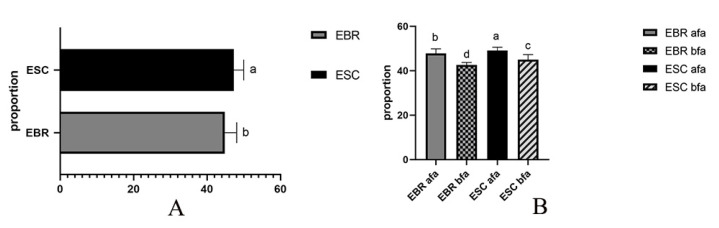
Comparison of rostral cuticle thickness in *Eucryptorrhynchus scrobiculatus* (ESC) females and *E. brandti* (EBR) females. (**A**) Comparison of overall rostral cuticle thickness; (**B**) comparison of rostral cuticle thickness before and after antennae. afa, after the antennal fossa; bfa, before the antennal fossa. (mean ± SE, *p* < 0.05, different letters “a” “b” “c” “d” indicate statistically significant differences).

The three-dimensional reconstruction of the exoskeleton of the rostrum and the head shows that the rostrum of *E. scrobiculatus* and *E. brandti* is hollow, cylindrical, and curved, and an extension of the near-spherical exoskeleton with a chewing mouthpart at the top. We define the hollow exoskeleton as the position from where the sub-spherical extends to the position where the chewing mouthparts appear as the “rostrum tube.” This segment of the exoskeleton forms our research object for studying the *E. scrobiculatus* and *E. brandti* rostrum. As shown in Figure 5 and Figure 6, for both *E. scrobiculatus and E. brandti*, the whole rostrum tube becomes thinner from the junction with the head cavity to the vicinity of the mouthparts, but the thickness irregularly changes. Through an analysis of the thickness of the rostrum tube cuticle, it was found that the thickness of the rostrum tube cuticle changes with the extension of the exoskeleton (Figure 5 and Figure 6). The proportion of the whole rostrum tube cuticle in *E. scrobiculatus* was significantly larger than that of *E. brandti* (Figure 7A). In addition, the proportion of the rostrum tube cuticle before and after the antennal fossa was significantly different, and the proportion of the rostrum tube cuticle after the antennal fossa was significantly greater than that before the antennal fossa in both *E. scrobiculatus and E. brandti*, ESC afa (49.07 ± 0.09) > EBR afa (47.77 ± 0.13) > ESC bfa (45.04 ± 0.14) > EBR bfa (42.64 ± 0.07), *p* < 0.05 (Figure 7B).

## 4. Discussion

Mouthparts are the feeding organs of insects. Due to the complex differentiation of insect feeding habits, they have formed various forms and functions to adapt to environmental changes, especially when new food sources appear [26]. The mouthparts of insects can be divided into several types by function, such as chewing mouthparts, piercing-sucking mouthparts, siphoning mouthparts, sponging mouthparts, cutting sponging mouthparts, etc. However, these mouthparts generally fall into two categories: those adapted for biting and chewing solid food, and those adapted for sucking up fluids [27,28]. Among beetles, the mouthparts of most beetles are designed to chew solid food. However, many beetles from the superfamily Curculionoidea have evolved unique rostra with mouthparts at the top that allow for feeding, as well as drilling and preparation of oviposition sites [1,2,7]. The mouthparts of weevils play an indispensable role in feeding, drilling, and ovipositing [6,7,29]. Howden suggested that the weevil’s oviposition methods can be classified into eleven different types, at least eight of which require the rostrum to assist with oviposition [30]. The morphological structure of the rostrum plays an important role in the oviposition of weevils. The apical evolution of weevils is a key innovation that enables this species to feed and lay eggs in almost all plant tissues, resulting in different life histories and great diversity. To make better use of the rostrum in the preparation of the oviposition hole, the female rostra of Attelabidae were further modified by fusing the gular sutures and reducing the ligula [2]. Through fusing the labrum and clypeus and developing advanced mandibles with long pharyngeal processes, the rostrum of Belidae can be transformed into a suitable oviposition tool to deposit their eggs into firm plant tissues, where their larvae endogenously develop [2].

The weevils of *Curculio* genus are an ideal target for assessing ecological morphological adaptation, since approximately 345 morphologically diverse species of the genus are found on a variety of host plants. The clearest phenotypic differences are in the size and shape of the rostra [31]. A study of 31 weevil species found that the wide diversity of host plant species of *Curculio* is thought to be caused by ecological morphological adaptations to oviposition sites, and host seed size is thought to be responsible for morphological changes in rostrum size [31]. The female rostra of *Rhopalapion longirostre* have a smooth surface and are suitable for drilling a long borehole through thick sepals, while the male rostra lack these adaptive structures and cannot deeply bore into the bud tissue [32]. Combined with the difference in oviposition behavior and sites of the two weevils, we speculate that the different rostrum surface of *E. scrobiculatus* and *E. brandti* are a manifestation of their application of different oviposition sites and substrate. The rough and uneven rostrum surface of *E. scrobiculatus* means that a continuous adhesion water film cannot form on the contact surface with the soil, reducing the adhesion of the soil to the rostrum surface. Moreover, in the process of digging in the soil, a large number of bristles on the rostrum deform and vibrate, which was not conducive to soil adhesion. These structures were beneficial for *E. scrobiculatus* females to excavate oviposition holes in the soil. The entire rostrum of *E. brandti* females curves smoothly downward at the same curvature. Their rostrum surface is smooth, with relatively few bristles and grooves, which is more conducive to drilling through the bark and laying eggs on the trunk of *A. altissima*.

In our study, the morphological characteristics of the whole mouthparts of *E. brandti* are similar to those of other weevil’s mouthparts. They are all chewing mouthparts, including the labrum, mandibles, maxillae, labium, and hypopharynx [7,24,25,28,33,34]. Some studies show the maxillae structure is uniquely modified in Curculionoidea [7,25,35]. Previous studies found that the maxillary palp of weevils have different numbers of segments; some are three-segmented palpi, while others are four-segmented palpi [25,33,36]. In our study we determined the difference in the number of segments of maxillary palp in *E. scrobiculatus* and *E. brandti* was due to different angles of observation. There are only three segments in the ventral view, but there are four segments in the dorsal view (Figure 2A–D). A morphological observation found that there are differences between the tips of the two weevils’ rostra. The maxillae and labium of *E. scrobiculatus* are encased by the mandibles, while the maxillae and labium of *E. brandti* are not completely enclosed by mandibles. Studies of oviposition behavior show that both weevils need to use antennae and the rostrum apex to find a suitable oviposition position before laying eggs [21,22]. The soil was soft, *E. brandti* used the mandibles to dig out part of the soil, and then used the maxillae and labium to probe to find a suitable oviposition position. *E. brandti* lay eggs on the trunk, and the females directly used the maxillae and labium exposed on the outside of the mandibles to probe a suitable oviposition position. The structural differences in the apex of the rostrum of the two weevils accommodates their respective oviposition patterns.

The feeding and oviposition of adult weevils are not restricted to the drilling of holes. Irregular excavations are also made, and some surface browsing is carried out, and these behaviors are inseparable from the role of the sensor. A comparison of the fine structures of maxillae and labium in two weevils revealed many sensilla distributed at the apex of the maxillae and labium, which can be divided into three types: sensilla basiconica (S.b.1–4), sensilla twig basiconica (S.tb.1–3), and digitiform sensilla (D.s). There was no difference in the number and distribution of sensilla basiconica between the two weevils at the apex of the maxillae and labium. These sensilla basiconica can be divided into two types: uniporous peg sensilla (S.b.2 and S.b.4) and porous peg sensilla (S.b.1 and S.b.3). The former may be a contact chemosensory organ, while the latter may function as an olfactory organ [25,37,38]. The latter acts as an olfactory receptor to cope with a variety of olfactory stimuli [39]. Faucheux speculates that the sensor in a woodboring beetle *Phoracanthe recurve* can sense water, sugar, amino acids and GABA [40]. In addition, Stadler et al. believe that cone sensors with a single hole at the top may also have a taste function [41]. The sensilla twig basiconica is similar to gustatory receptors. This sensilla with similar morphometrics can also be found on the maxillary and labial palps of *Chrysolina aeruginosa* Fald., *Ips subelongatus* [42,43]. In addition to olfactory function, this sensilla may have mechanical receptors and play a certain role in ovipositing [44,45]. Yang suggest that some sensilla twig basiconica might function in the reception of olfactory signals, and some sensilla twig basiconica might function as hygro-/thermo-receptors [23]. Therefore, these sensilla may play an important role in searching and locating the oviposition sites of *E. scrobiculatus* and *E. brandti*. In our study, S.tb.3 was found only at the apex of labial palpus of *E. brandti* females, whereas, for the other sensilla types, structure and numbers were not significantly different between *E. scrobiculatus and E. brandti* females. Research of the oviposition behavior of the two weevils shows that during the oviposition process, gravid females use antennae and mouthparts to probe the suitability of a site for egg laying [21]. *E. scrobiculatus* females laid eggs in the soil near *A. altissima*, while *E. brandti* females laid eggs in *A. altissima* trunks. Combined with the study of oviposition behavior and sites, we inferred S.tb.1 and S.tb.2 played a role in searching and locating oviposition sites. S.tb.3 may be able to identify the host and judge whether the position between the host phloem and xylem is a suitable oviposition site. Digitiform sensilla (D.s) were only ventrally observed in the last segment of the maxillary palp. The sensor structure is similar to the neuronal structure of hygro-/thermo-receptors [46,47]. Therefore, the digitiform sensilla of *E. scrobiculatus* and *E. brandti* females likely play roles in monitoring the environmental conditions in the process of ovipositing.

**Figure 8:**
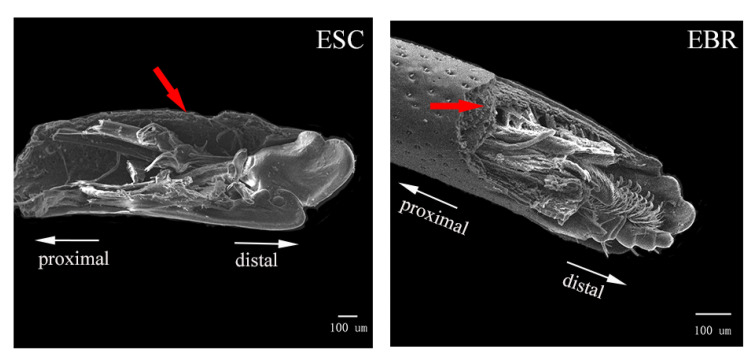
The lamellar microstructure of *Eucryptorrhynchus scrobiculatus* (ESC) females and *E. brandti* (EBR) females under scanning electron microscope. The red arrows indicate laminate structures.

The rostral cuticle structure of *E. scrobiculatus* and *E. brandti* females were reconstructed by micro-CT. It was difficult to distinguish the inner and outer epidermis of the rostrum in micro-CT sections; therefore, we studied the general name of the inner and outer epidermis in the cuticle structure. Through the study of the rostral cuticle, it was found that the thickness of the exoskeleton from the head cavity junction to the appearance of the chewable mouthpart was constantly changing. Taking the antennal fossa as the dividing line, the proportion of cuticles after the antennal fossa was significantly greater than that before the antennal fossa. The proportion of cuticle thickness in *E. scrobiculatus* females in the same position was always higher than that of *E. brandti* females, regardless of whether it was before and after the antennal fossa or the overall rostral cuticle (Figure 7A). Therefore, we inferred that in both *E. scrobiculatus* and *E. brandti*, the rigidity of the rostrum before the antennae fossa was smaller than that after the antennae fossa, but the toughness is greater than that after the antennae fossa. The overall rigidity of the *E. brandti* rostrum is less than that of *E. scrobiculatus*, but the toughness is larger than that of *E. scrobiculatus*. Such differences in rostrum structure are more conducive to the preparation of oviposition sites by *E. scrobiculatus* in the soil and *E. brandti* on the trunk. Previous studies showed that the exoskeleton of Coleoptera (Beetle) was a hierarchical fiber complex characterized by various arrangements embedded in heterogeneous protein matrices α-chitin (N-acetylglucosamine) nanofibers [48,49,50,51]. Although α-chitin had brittleness and strong anisotropy, it also had rigidity and toughness due to the unique lamellar microstructure of the beetle cuticle [51,52,53,54]. In this study, we attempted to use the semi-thin slice method to explore whether the epidermis of the two weevils had this structure. However, due to the brittleness of its exoskeleton, the sample cannot be formed during the cutting process, and the desired results were not achieved, even after several experiments. Fortunately, we found this laminate structure in the rostral cuticle structure of *E. brandti* by scanning electron microscopy (Figure 8). This structure has previously been demonstrated in Acron weevils, to avoiding the catastrophic bending of the rostrum when feeding and excavating oviposition cavity [51]. Therefore, we suppose that the thickness change and lamination structure of the rostral cuticle of *E. scrobiculatus* and *E. brandti* are adaptations to the oviposition position, which can avoid the failure of the rostrum during excavation.

Previous studies showed that *E. scrobiculatus* and *E. brandti* may be derived from the same ancestor [55]. Because climate change leads to a decrease in host resources, increase in competition within the population, and niche differentiation, groups occupying different niches have genetic communication barriers, and new species are formed through reproductive isolation [55]. In other words, two closely related species of *E. scrobiculatus* and *E. brandti* were formed. *E. scrobiculatus* and *E. brandti* females must dig an oviposition hole with their rostra before laying eggs [20,21]; the two weevils utilized different oviposition sites to facilitate coexistence on the single host *A. altissima* [20]. The differences in the morphology of mandibles and the control of the adductor muscles of the two weevils indicated that they had different bite forces and adapt to laying at different oviposition sites [56]. On this basis, combined with the oviposition behavior of the two weevils, this study compared the differences in the fine structures of the rostra of the two weevils. We also discussed the adaptation of the rostra of the two weevils to their oviposition position from the perspective of the differences in the distribution of sensilla and the cuticle structure of the rostra tube. This study is a supplement to previous research on the adaptation of the rostra structure of the two weevils to the ecological demands of egg deposition. Our findings also provide a more theoretical basis for explaining the coexistence of the two weevils on the same host *A. altissima*.

## 5. Conclusions

In this study, the differences in the fine structures of the rostra between *E. scrobiculatus* and *E. brandti* were compared. The rostrum surface of *E. scrobiculatus* is rougher, the proportion of the whole rostrum tube cuticle is larger, the maxillae and labium are encased by the mandibles. These structures make *E. scrobiculatus* more suitable for excavating cavities in the soil. The rostrum surface of *E. brandti* is smoother, the proportion of the whole rostrum tube cuticle is smaller, the maxillae and labium are not completely enclosed by mandibles. These structures make *E. brandti* more suitable for excavating cavities on the trunk of host. In addition, the thickness change and the unique laminate structure in the rostral cuticle structure of both *E. scrobiculatus* and *E. brandti* give their rostra tubes a good rigidity and toughness, which avoids the phenomenon of rostrum fracture in the process of excavating. In summary, these features play an important role in exploring the oviposition mechanism of the weevil from the perspective of structure and function, but also provide new insights into the coexistence of two weevil species in the same host *A. altissima*.

## Data Availability

The data presented in this study are available on request from the corresponding author.

## References

[1] Anderson R. (1995). An evolutionary perspective on diversity in Curculionoidea. Mem. Entomol. Soc. Washington.

[2] Oberprieler R.G., Marvaldi A.E., Anderson R.S. (2007). Weevils, weevils, weevils everywhere. Zootaxa.

[3] Hanley R.S., Goodrich M.A. (1995). Review of mycophagy, host relationships and behavior in the New World oxyporinae (Coleoptera: Staphylinidae). Coleopt. Bull..

[4] Moon M.J., Kim H., Park J.G., Choi W.I. (2014). Mouthparts of the bark beetle (*Ips acuminatus*) as a possible carrier of pathogenic microorganisms. J. Asia-Pacif. Entomol..

[5] Toki W., Togashi K. (2013). Relationship between Oviposition Site Selection and Mandibular Asymmetry in Two Species of Lizard Beetles, *Anadastus pulchelloides* Nakane and *Doubledaya bucculenta* Lewis (Coleoptera: Erotylidae: Languriinae). Coleopt. Bull..

[6] Van Zandt P.A., Townsend V.R., Carlton C.E., Blackwell M., Mopper S. (2003). *Loberus impressus* (LeConte) (Coleoptera: Erotylidae) Fungal Associations and Presence in the Seed Capsules of *Iris Hexagona*. Coleopt. Bull..

[7] Moon M.J., Park J.G., Kim K.H. (2008). Fine structure of the mouthparts in the ambrosia beetle *platypus koryoensis* (Coleoptera: Curculionidae: Platypodinae). Anim. Cells Syst..

[8] Zwolfer H. (1975). Ruesselkaefer und ihre Umwelt-ein Kapitel Okologie. Weevils and environment—A chapter about ecology. Stuttg. Beitr. Naturk..

[9] Zimmerman E. (1994). Anthribidae to attelabidae: The primitive weevils. Australian Weevils (Coleoptera: Curculionoidea) I.

[10] Zimmerman E. (1994). Brentidae, eurhynchidae, apionidae and a chapter on immature stages by Brenda May. Australian Weevils (Coleoptera: Curculionoidea) II.

[11] Burke H.R. (1993). Australian weevils (Coleoptera: Curculionoidea). Ann. Entomol. Soc. Am..

[12] Zhao Y., Chen Y. (1980). China Economic Insects (Coleoptera: Curculionidae).

[13] Yang P. (2015). Preliminary Study of the Artificial Raising and Prevention of *Eucryptorrhynchus chinensis*. Master’s Thesis.

[14] Wen X.J., Yang K.L., Wen J.B. (2016). Host-independent artificial rearing of *Eucryptorrhynchus brandti* (Coleoptera: Curculionidae). Biocontrol Sci. Technol..

[15] Yu Q.Q. (2013). Preliminary Study on Biology and Artificial Rearing of *Eucryptorrhynchus chinensis* (Olivier) (Coleptera: Curculionidae). Master’s Thesis.

[16] Ding J., Wu Y., Zheng H., Fu W., Reardon R., Liu M. (2006). Assessing potential biological control of the invasive plant, tree-of-heaven, *Ailanthus altissima*. Biocontrol Sci. Technol..

[17] Herrick N.J., Mcavoy T.J., Snyder A.L., Salom S.M., Kok L.T. (2012). Host-range testing of *Eucryptorrhynchus brandti* (Coleoptera: Curculionidae), a candidate for biological control of tree-of-heaven, *Ailanthus altissima*. Environ. Entomol..

[18] Herrick N.J., Salom S.M., Kok L.T., Mcavoy T.J. (2009). Foliage feeding tests of *Eucryptorrhynchus brandti* (Harold) (Coleoptera: Curculionidae), a potential biological control agent of the tree-of-heaven, *Ailanthus altissima*. USDA Res. Forum Invasive Spec..

[19] Mcavoy T.J., Salom S.M., Yu B., Ji H.L., Du Y.Z., Johnson N., Reardon R., Kok L.T. (2014). Occurrence and development of *Eucryptorrhynchus brandti* and *E. chinensis* (Coleoptera: Curculionidae) on *Ailanthus altissima* trees subjected to different levels of mechanical damage. Biocontrol Sci. Technol..

[20] Zhang G.Y., Ji Y.C., Gao P., Wen J.B. (2019). Oviposition Behavior and Distribution of *Eucryptorrhynchus scrobiculatus* and *E. brandti* (Coleoptera: Curculionidae) on *Ailanthus altissima* (Mill.). Insects.

[21] Zhang G.Y., Ji Y.C., Wen X.J., Li Q., Ren Y., Wen J.B. (2017). Oviposition behaviour of *Eucryptorrhynchus brandti* (Coleoptera: Curculionidae: Cryptorrhychinae) on *Ailanthus altissima* (Mill.) Swingle (Sapindales: Simaroubaceae). Biocontrol Sci. Technol..

[22] Zacharuk R.Y. (1980). Ultrastructure and function of insect chemosensilla. Annu. Rev. Entomol..

[23] Yang Y.C., Ren L.L., Wang T., Xu L.L., Zong S.X. (2017). Comparative morphology of sensilla on antenna, maxillary palp and labial palp of larvae of *Eucryptorrhynchus scrobiculatus* (Olivier) and *E. brandti* (Harold) (Coleoptera: Curculionidae). Acta Zool..

[24] Belhoucine L., Bouhraoua R.T., Prats E., Pulade-Villar J. (2013). Fine structure and functional comments of mouthparts in *Platypus cylindrus* (Col., Curculionidae: Platypodinae). Micron.

[25] Chen F., Zhang C.N., Dai W. (2016). Fine Structure and Sensory Apparatus of the Mouthparts of the Maize Weevil, *Sitophilus zeamais* Motschulsky (Coleoptera: Curculionoidea: Dryophthoridae). Ann. Entomol. Soc. Am..

[26] Jervis M. (1998). Functional and evolutionary aspects of mouthpart structure in parasitoid wasps. Biol. J. Linn. Soc..

[27] Gullan P., Cranston P. (2010). The Insects: An Outline of Entomology.

[28] Moon M. (2015). Microstructure of mandibulate mouthparts in the greater rice weevil, *Sitophilus zeamais* (Coleoptera: Curculionidae). Entomol. Res..

[29] Atkinson T.H. (2000). Ambrosia Beetles, *Platypus* spp. (Insecta: Coleoptera: Platypodidae). DPI Entomol. Circ. Univ. Florida.

[30] Howden A. (1995). Structures related to oviposition in Curculionoidea. Mem. Ent. Soc. Wash..

[31] Joseph H., Alfried P.V. (2004). Ecomorphological adaptation of acorn weevils to their oviposition site. Evolution.

[32] Wilhelm G., Handschuh S., Plant J., Nemeschkal H.L. (2011). Sexual dimorphism in head structures of the weevil *Rhopalapion longirostre* (Olivier 1807) (Coleoptera: Curculionoidea): A response to ecological demands of egg deposition. Biol. J. Linn. Soc..

[33] Morimoto K., Hiroaki K. (2003). Morphological characters of the weevil head and phylogenetic implications (Coleoptera, Curculionoidea). Esakia.

[34] Davis S.R. (2011). Delimiting baridine weevil evolution (Coleoptera: Curculionidae: Baridinae). Zool. J. Linn. Soc..

[35] Farrell B.D., Sequeira A.S., O’Meara B.C., Normark B.B., Jordal B.H. (2001). The evolution of agriculture in beetles (Curculionidae: Scolytinae and Platypodinae). Evolution.

[36] Bae J.D., Park S.O., Lee J.E. (2000). Comparative morphology of the mouthparts of the Curculionoidea (Coleoptera), their feeding mechanism and relationship to classification. Part I. Family Brentidae. Korean J. Environ. Biol..

[37] Faucheux M.J. (1991). Morphology and distribution of sensilia on the cephalic appendages, tarsi and ovipositor of the European sunflower moth, *Homoeosoma nebulella* Den. & Schiff. (Lepidoptera: Pyralidae). Int. J. Insect Morphol. Embryol..

[38] Faucheux M.J. (1995). Sensilla on the antennae, mouthparts, tarsi and ovipositor of the sunflower moth, *Homoeosoma electellum* (Hulster) (Lepidoptera, Pyralidae): A scanning electron microscopic study. Ann. Sci. Nat. Zool. Biol. Anim..

[39] Doane J.F., Klingler J. (1978). Location of CO2-Receptive Sensilla on Larvae of the Wireworms *Agriotes lineatus-obscurus* and *Limonius californicus*. Ann. Entomol. Soc. Am..

[40] Faucheux M.J. (2013). Mouthpart sensilla of the adult yellow longicorn beetle *Phoracantha recurva* Newman, 1840 (Coleoptera, Cerambycidae, Cerambycinae). Bull. De L’institut Sci. Rabat Sect. Sci. De La Vie..

[41] Staedler E., Seabrook W. (1975). Chemoreceptors on the proboscis of the female eastern spruce budworm: Electrophysiological study. Entomol. Exp. Appl..

[42] Zhang L., Ren L.L., Luo Y.Q., Zong S.X. (2013). Scanning electron microscopy analysis of the cephalic sensilla of *Chrysolina aeruginosa* Fald. (Coleoptera, Chrysomelidae). Microsc. Res. Tech..

[43] Shi X., Zhang S.F., Liu F., Xu F.Y., Zhang F.B., Bin Guo X., Zhang Z., Kong X.B. (2021). SEM analysis of sensilla on the mouthparts and antennae of Asian larch bark beetle *Ips subelongatus*. Micron.

[44] Xu L., Xie C., Zheng H., Liu Y. (2019). Scanning electron microscopic observation of ovipositor sensilla of *Sclerodermus guani* (Hymenoptera: Bethylidae). J. Jiangsu For. Sci. Technol..

[45] Zhang L. (2015). Comparative Study on Ovipositor Sensilla of Insects with Four Different Types of Oviposition Strategies. Master’s Thesis.

[46] Ortloff A., Zanetti N., Centeno N., Silva R., Bustamante F., Olave A. (2014). Ultramorphological characteristics of mature larvae of *Nitidula carnaria* (Schaller 1783) (Coleoptera: Nitidulidae), a beetle species of forensic importance. Forensic Sci. Int..

[47] Eilers E.J., Talarico G., Hansson B.S., Hilker M., Reinecke A. (2012). Sensing the underground—Ultrastructure and function of sensory organs in root-feeding *Melolontha melolontha* (Coleoptera: Scarabaeinae) larvae. PLoS ONE.

[48] Jansen M.A., Singh S.S., Chawla N., Franz N.M. (2016). A multilayer micromechanical model of the cuticle of *Curculio longinasus* Chittenden, 1927 (Coleoptera: Curculionidae). J. Struct. Biol..

[49] Vincent J.F., Wegst U.G. (2004). Design and mechanical properties of insect cuticle. Arthropod Struct. Dev..

[50] Hepburn H., Ball A. (1973). On the structure and mechanical properties of beetle shells. J. Mater. Sci..

[51] Jansen M.A., Williams J., Chawla N., Franz N.M. (2019). Avoidance of Catastrophic Structural Failure as an Evolutionary Constraint: Biomechanics of the Acorn Weevil Rostrum. Adv. Mater..

[52] Van de Kamp T., Riedel A., Greven H. (2016). Micromorphology of the elytral cuticle of beetles, with an emphasis on weevils (Coleoptera: Curculionoidea). Arthropod Struct. Dev..

[53] Van de Kamp T., Greven H. (2010). On the architecture of beetle elytra. Entomol. Heute.

[54] Neville A., Parry D., Woodhead-Galloway J. (1976). The chitin crystallite in arthropod cuticle. J. Cell Sci..

[55] Liu Z.K. (2016). Molecular Phylogeny and Species Differentiation of the *Eucryptorrhynchus scrobiculatus* and *E. brandti*. Ph.D. Thesis.

[56] Zhang G.Y., Guo W.J., Wang X.Y., Wang Q., Cui J., Wen J.B. (2021). Structural comparison of the rostra of two species of weevils coexisting on *Ailanthus altissima*: The response to ecological demands of egg deposition. BMC Ecol. Evol..

